# Supplementary material for: Nicotine Exposure in a Phencyclidine-Induced Mice Model of Schizophrenia: Sex-Selective Medial Prefrontal Cortex Protein Markers of the Combined Insults in Adolescent Mice
Source: Int J Mol Sci. 2023 Sep 27;24(19):14634. doi: 10.3390/ijms241914634 (PMC10572990; doi:10.3390/ijms241914634)
Supplement: Supplementary file 1 [file ijms-24-14634-s001.zip › Rodríguez-VegaSM2-Results.pdf]

SUPPLEMENTARY MATERIAL 2

International Journal of Molecular Sciences

Nicotine exposure in a phencyclidine-induced mice model of schizophrenia: Sex-selective medial prefrontal cortex protein markers of the combined insults in adolescent mice

Andrés Rodríguez-Vega[1], Ana Carolina Dutra-Tavares[1], Thainá Pereira de Souza[1], Keila A. Semeão[1], Claudio C. Filgueiras[1], Anderson Ribeiro-Carvalho[2], Alex C. Manhães[1], Yael Abreu-Villaça[1*]

1. Laboratório de Neurofisiologia, Departamento de Ciências Fisiológicas, Instituto de Biologia Roberto Alcantara Gomes, Universidade do Estado do Rio de Janeiro (UERJ), Av. Prof. Manuel de Abreu 444, 5 andar – Vila Isabel, Rio de Janeiro, RJ, 20550-170, Brazil.
2. Departamento de Ciências, Faculdade de Formação de Professores da Universidade do Estado do Rio de Janeiro, São Gonçalo, RJ, 24435-005, Brazil.

Corresponding author: Dr. Yael Abreu-Villaça
E–mail: yael_a_v@yahoo.com.br
yael_a_v@pq.cnpq.br
ORCID: 0000-0002-9801-6179

Short title: Adolescent nicotine and schizophrenia mPFC proteomic profile

Proteomic profile of the mPFC of PCP and NIC mice when compared to controls

Figure S1 shows results from the proteomic analysis of the mPFC of PCP, NIC, and PCPNIC mice when compared to CT. The number of deregulated proteins in PCP mice (males = 38, females = 36) was smaller than that identified in NIC (males = 134, females = 68).

The impact of nicotine (Fig. S1 A) was characterized by 23 proteins differentially expressed exclusively in NIC males (15 upregulated and 8 downregulated) and 21 proteins in NIC females (14 upregulated and 7 downregulated) when compared to CT. Among the top 6 enriched pathways, 3 (immune system, vesicle-mediated transport, and membrane trafficking) were affected in both males and females (Fig. S1 B). In males, metabolism, metabolism of proteins, and post-translational protein modification also stood out. Distinctively, in females, the most relevant pathways involved signal transduction and signaling by Rho GTPases, Miro GTPases, and RHOBTB3 (Fig. S1 B).

Regarding the impact of phencyclidine (Fig. S1 A), there were 14 proteins differentially expressed exclusively in PCP males (5 upregulated and 9 downregulated) and 8 proteins in PCP females (4 upregulated and 4 downregulated). Three of the top 6 enriched Reactome pathways were common to males and females (immune system, signal transduction, and signaling by receptor tyrosine kinases) (Fig. S1 C). In males, post-translational modification and metabolism of proteins and RAB geranylgeranylation aggregated more proteins. In females, those were hemostasis-related pathways (Fig. S1 C).

A detailed list of deregulated proteins involved in each comparison can be found in Supplementary Material, Sheets 3 to 8.

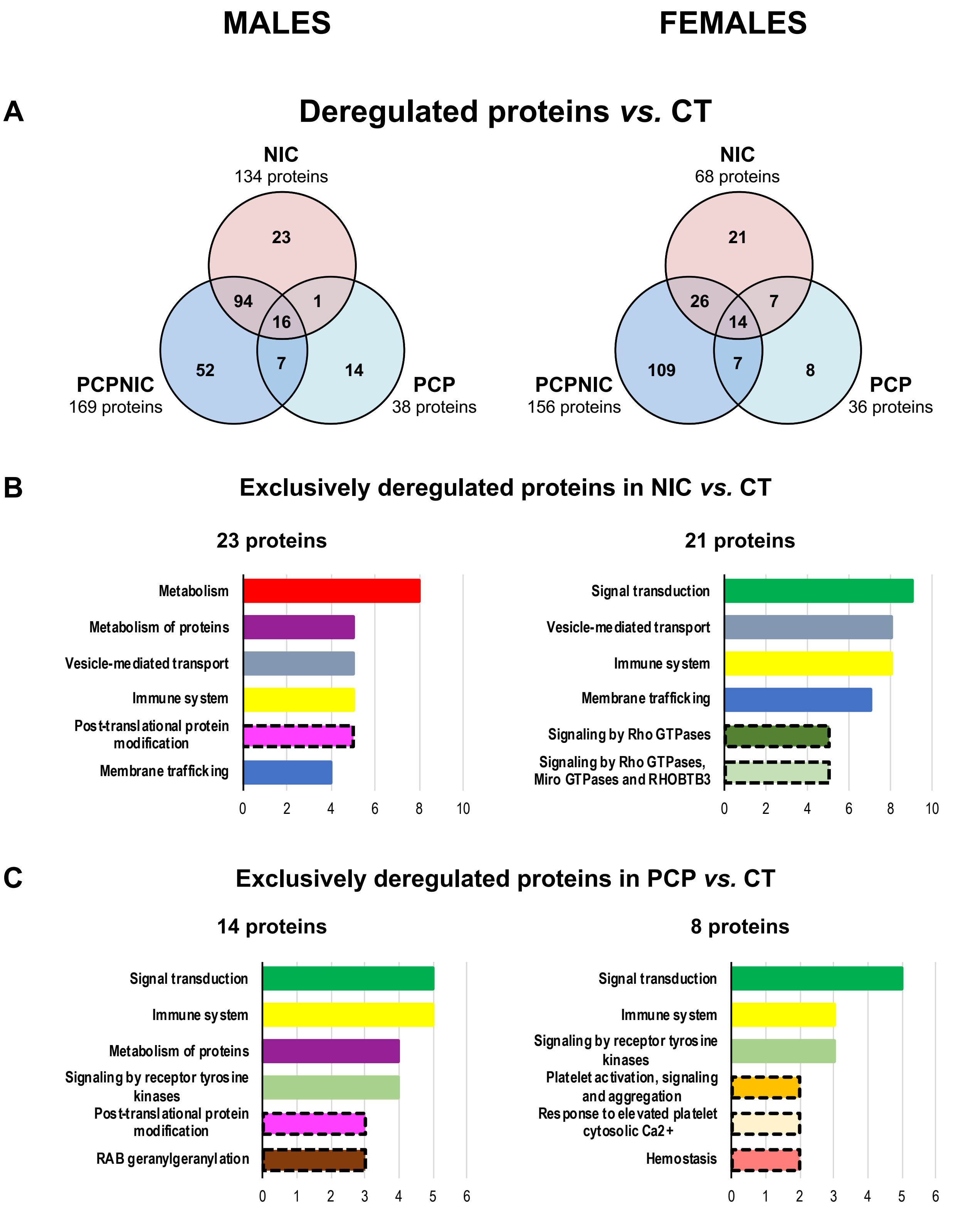

Figure S1. Proteomic profile of the mPFC of male (left) and female (right) mice exposed to phencyclidine (PCP), nicotine (NIC), or the combined insult (PCPNIC) when compared to controls (CT). A: Venn diagrams showing deregulated proteins in NIC, PCP, and PCPNIC mice when compared to CT ones. B: Top 6 Reactome pathways of exclusively deregulated proteins in NIC when compared to CT. C: Top 6 Reactome pathways of exclusively deregulated proteins in PCP when compared to CT. Each color bar graph represents a specific pathway. Dashed outlines represent pathways identified only once.

Proteomic profile of the mPFC of PCP mice when compared to NIC ones

In total, in males, 114 proteins were differentially expressed in PCP as compared to NIC mice. In females, 24 proteins were deregulated in PCP mice.

Proteins that were not targeted by nicotine history were those exclusively deregulated in PCP as compared to NIC mice. (Fig. S2 A). In males, the analysis indicated 84 proteins, and in females, 12 proteins were exclusively deregulated in PCP mice. Also, there were 30 proteins commonly deregulated in PCPNIC and PCP males and 12 proteins commonly deregulated in females (Fig. S2 A).

The Reactome analysis of the exclusively deregulated proteins in PCP mice (Fig. S2 B) indicated immune system, signal transduction, and cellular responses to stress as top pathways for both males and females. In males, other relevant pathways involved metabolism, membrane trafficking, and vesicle-mediated transport. Distinctively, in females, those were related to the innate immune system, transport of small molecules, and cellular responses to stimuli (Fig. S2 B).

A detailed list of the proteins involved in each comparison can be found in Supplementary Material, Sheets 9 to 12.

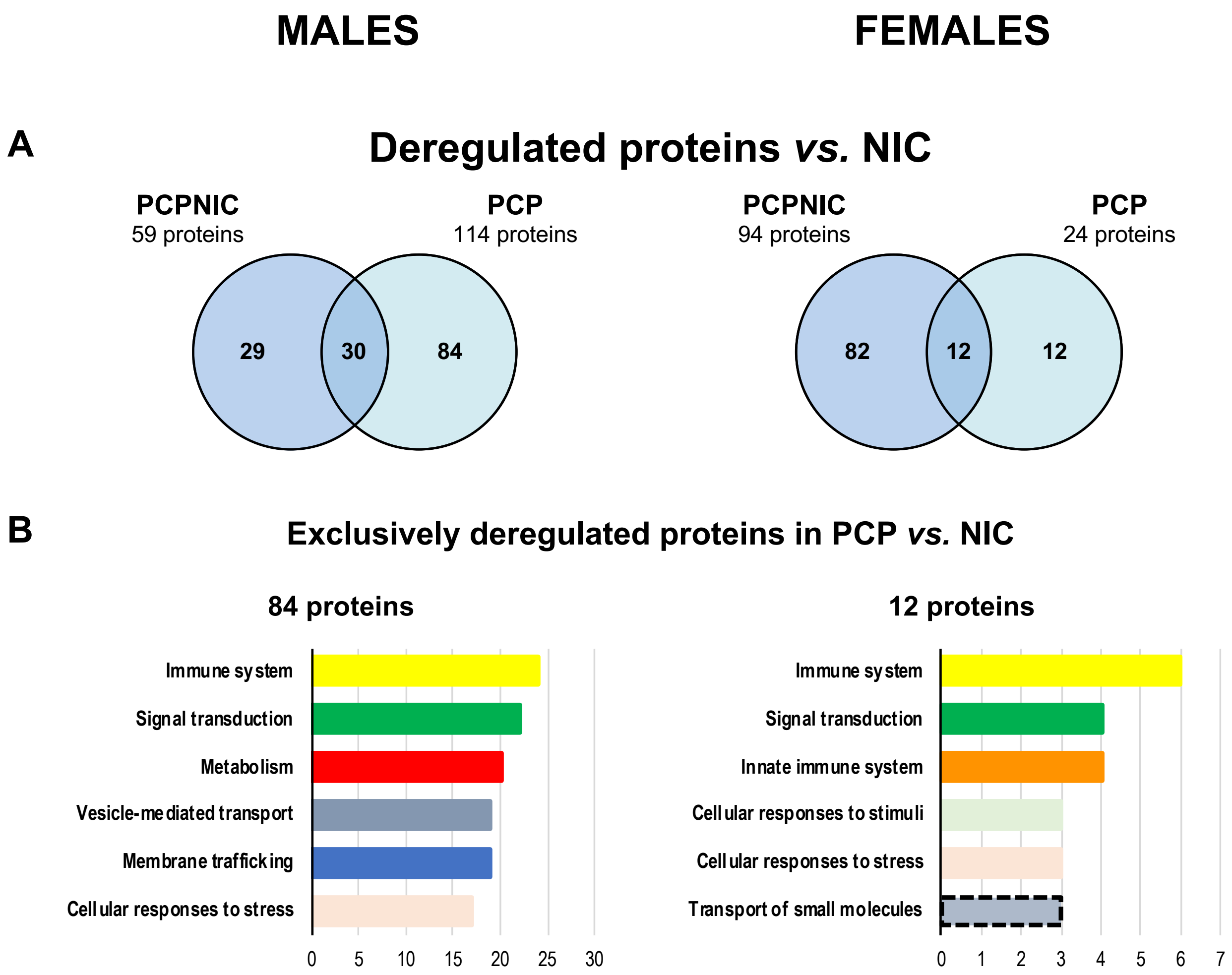

Figure S2. Proteomic profile of the mPFC of male (left) and female (right) mice exposed to phencyclidine (PCP) or the combined insult (PCPNIC) when compared to nicotine-exposed (NIC) ones. **A**: Venn diagrams showing deregulated proteins in PCP and PCPNIC mice when compared to NIC ones. **B**: Top 6 Reactome pathways of exclusively deregulated proteins in PCP when compared to NIC. Each color bar graph represents a specific pathway. Dashed outlines represent pathways identified only once.

Protein markers in the mPFC of NIC and PCP mice

To identify protein markers of NIC exposure in males and females, Venn diagrams of commonly deregulated proteins in NIC when compared to CT (males = 23, females = 21), in NIC when compared to PCP (males = 84, females = 12), and in the comparison between NIC and PCP-NIC mice (males = 29, females = 82) were designed (Fig. S3 A). A similar approach was adopted aiming to identify protein markers of SCHZ: commonly deregulated proteins in PCP when compared to CT (males = 14, females = 8), in PCP when compared to NIC (males = 84, females = 12), and in the comparison between PCP and PCPNIC mice (males = 142, females = 84) are presented as Venn diagrams (Fig. S3 B). We failed to identify specific markers of nicotine exposure in both males and females. In males modeled to SCHZ, 3 proteins were identified and, in females, 1 protein (Fig. S3 B, Tables S1 and S2). WNT signaling-related pathways were enriched in both males (WNT5A-dependent internalization of FZD proteins and signaling by nuclear receptors) and females (beta-catenin independent WNT signaling, signaling by WNT and Ca^{++} pathway) (Fig. S3 C). In males, other pathways included signal transduction, recycling pathway of L1, clathrin-mediated endocytosis, and platelet activation. More details about these proteins can be found in Supplementary Material, Sheets 15, and 16.

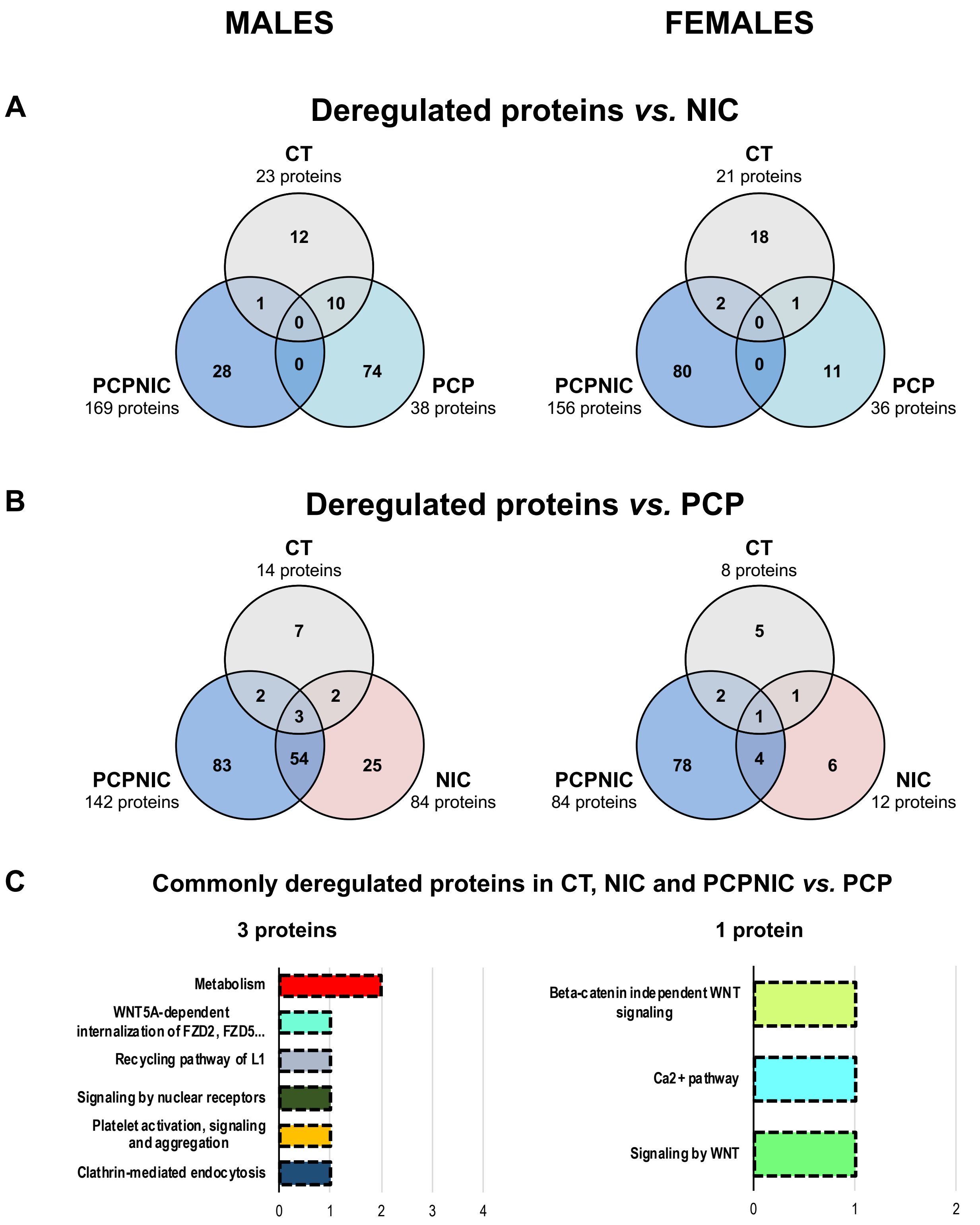

Figure S3. Proteomic profile of the mPFC of male (left) and female (right) mice exposed to nicotine (NIC, A) and to phencyclidine (PCP, B and C) when compared to all other groups. **A**: Venn diagrams showing deregulated proteins in NIC mice when compared to CT, PCP and PCPNIC ones. **B**: Venn diagrams showing deregulated proteins in PCP mice when compared to CT, NIC and PCPNIC ones. **C**: Top 6 Reactome pathways of commonly deregulated proteins in PCP when compared to CT, NIC and PCPNIC ones. Each color bar graph represents a specific pathway. Dashed outlines represent pathways identified only once.

Table S1. Protein markers of PCP male mice

Description	Gene	**PCP** *vs.* CT	**PCP** *vs*. NIC	**PCP** *vs.* PCPNIC
Clathrin light chain A	Clta	Down	Up	Up
Guanine nucleotide-binding protein G(t) subunit alpha-3	Gnat3	Down	Down	Down
Glyceraldehyde-3-phosphate dehydrogenase_ testis-specific	Gapdhs	Down	Up	Up

Table S2. Protein markers of PCP female mice

Description	Gene	**PCP** *vs.* CT	**PCP** *vs*. NIC	**PCP** *vs.* PCPNIC
Guanine nucleotide-binding protein G(o) subunit alpha	Gnao1	Up	Up	Up
