# Supplementary material for: Nicotine Exposure in a Phencyclidine-Induced Mice Model of Schizophrenia: Sex-Selective Medial Prefrontal Cortex Protein Markers of the Combined Insults in Adolescent Mice
Source: Int J Mol Sci. 2023 Sep 27;24(19):14634. doi: 10.3390/ijms241914634 (PMC10572990; doi:10.3390/ijms241914634)
Supplement: Supplementary file 1 [file ijms-24-14634-s001.zip › Rodríguez-VegaSM3-Discussion.pdf]

SUPPLEMENTARY MATERIAL 3

International Journal of Molecular Sciences

Nicotine exposure in a phencyclidine-induced mice model of schizophrenia: Sex-selective medial prefrontal cortex protein markers of the combined insults in adolescent mice

Andrés Rodríguez-Vega[1], Ana Carolina Dutra-Tavares[1], Thainá Pereira de Souza[1], Keila A. Semeão[1], Claudio C. Filgueiras[1], Anderson Ribeiro-Carvalho[2], Alex C. Manhães[1], Yael Abreu-Villaça[1*]

1. Laboratório de Neurofisiologia, Departamento de Ciências Fisiológicas, Instituto de Biologia Roberto Alcantara Gomes, Universidade do Estado do Rio de Janeiro (UERJ), Av. Prof. Manuel de Abreu 444, 5 andar – Vila Isabel, Rio de Janeiro, RJ, 20550-170, Brazil.
2. Departamento de Ciências, Faculdade de Formação de Professores da Universidade do Estado do Rio de Janeiro, São Gonçalo, RJ, 24435-005, Brazil.

Corresponding author: Dr. Yael Abreu-Villaça
E–mail: yael_a_v@yahoo.com.br
yael_a_v@pq.cnpq.br
ORCID: 0000-0002-9801-6179

Short title: Adolescent nicotine and schizophrenia mPFC proteomic profile

Isolated sub-networks

mPFC interactome map of PCPNIC male mice

The only sub-network that was not linked to *Amph* in the male interactome map was the Energy metabolism, composed of 2 proteins: Phosphoglycerate kinase 2 (*Pgk2*) and Malate dehydrogenase cytoplasmic (*Mdh1*). *Pgk2* acts in the first ATP-generating step of the glycolytic pathway converting 1,3-diphosphoglycerate into 3-phosphoglycerate. In the gluconeogenic pathway, *Pgk2* catalyzes the reverse reaction. As for *Mdh1*, it catalyzes the reversible oxidation

of malate to oxaloacetate in the citric acid cycle and is a major participant in the malate-aspartate shuttle, a passage from the cytosol to the mitochondria essential for energy production. The presence of these proteins in the male interactome map corroborates previous studies that demonstrate impaired energy metabolism in SCHZ patients (Henkel et al., 2022; Martins-De-Souza et al., 2011). *Mdh1* is downregulated in the dorsolateral prefrontal cortex of SCHZ patients, which may decrease energy production via glycolysis (Martins-de-Souza et al., 2009; Middleton et al., 2002). Also, in a preclinical study that used MK-801 (an NMDA receptor antagonist) to model SCHZ, there were significant changes in the levels of glycolytic enzymes, including *Pgk*, with oligodendrocytes being more severely impacted (Guest et al., 2015).

mPFC interactome map of PCPNIC female mice

In the female interactome map, there were 2 isolated sub-networks. Septin-8 *(Sept8*) and Septin-10 *(Sept10*) formed the Septin cytoskeleton sub-network. Septins compose a conserved family of GTPases involved in various cellular processes, including synaptic vesicle trafficking, exocytosis, cell signaling, and apoptosis (Benoit et al., 2023; Peterson and Petty, 2010). Thirteen paralogs have been identified and clustered into 4 subgroups. Both *Sept8* and *Sept10* are components of the SEPT6 group, together with Septin 6, Septin 11 and Septin14 (Werner and Yadav, 2022). *Sept8* contributes to neurite development and branching (Werner and Yadav, 2022) and its depletion disrupts neurite elongation (Ageta-Ishihara and Kinoshita, 2021). It was shown to inhibit neurotransmitter release, as a result preventing overexcitation (Ageta-Ishihara and Kinoshita, 2021). In addition, septins including the SEPT6 subgroup, are enriched in the postsynaptic density (Ageta-Ishihara and Kinoshita, 2021; Werner and Yadav, 2022). These data corroborate previous reports that link altered cytoskeleton components to SCHZ and nicotine exposure (Ehlinger et al., 2017; Jung et al., 2016; Marchisella et al., 2016) and suggest a role of septin dysregulation.

Ras-related protein Rab-37 (*Rab37*) and HMG box transcription factor BBX (*Bbx)* composed the Cell cycle subnetwork. When considering trafficking pathways, the Rab GTPase family stands out. This family is composed of a very heterogeneous group of proteins that, through their effectors, regulate vesicle formation, actin- and tubulin-dependent vesicle movement, and membrane fusion (Stenmark and Olkkonen, 2001). *Rab37* gene promotes cell division but also the differentiation of neuronal cells (Hagag et al., 1986; Noda et al., 1985). These roles are closely related to those assigned to *Bbx. Bbx* is a member of the high mobility

group (HMG)-box proteins, a superfamily of architectural proteins (Chen et al., 2014). It is a sequence-specific transcription factor and is expressed in progenitor cells of the developing neocortex ventricular zone, promoting progenitor cell self-renewal (Dixon et al., 2013). Its role in cell cycle progression has also been shown in yeast, in which *Bbx* promotes the G1/S phase transition (Sanchez-Dias Az et al., 2001).

Besides previous evidence that associate the proteins that compose the Energy metabolism subnetwork of males (*Mdh1* and *Pgk2*) with SCHZ, no previous data describe specific contributions of the proteins that compose the female Septin cytoskeleton (*Sept8* and *Sept10)*, and Cell cycle (*Rab37* and *Bbx)* subnetworks to SCHZ or the comorbidity. Accordingly, possible deleterious outcomes of their disbalance await further investigation. Notwithstanding, the fact that these 3 sub-networks are isolated from the main nets of proteins described in the text of the manuscript suggests independent mechanisms of interference in the comorbidity.

References

88. Marchisella, F., Coffey, E.T., Hollos, P., 2016. Microtubule and microtubule associated protein anomalies in psychiatric disease. Cytoskeleton 73, 596–611. https://doi.org/https://doi.org/10.1002/cm.21300
94. Ehlinger, D.G., Burke, J.C., McDonald, C.G., Smith, R.F., Bergstrom, H.C., 2017. Nicotine-induced and D1-receptor-dependent dendritic remodeling in a subset of dorsolateral striatum medium spiny neurons. Neuroscience 356, 242–254. https://doi.org/https://doi.org/10.1016/j.neuroscience.2017.05.036
95. Jung, Y., Hsieh, L.S., Lee, A.M., Zhou, Z., Coman, D., Heath, C.J., Hyder, F., Mineur, Y.S., Yuan, Q., Goldman, D., Bordey, A., Picciotto, M.R., 2016. An epigenetic mechanism mediates developmental nicotine effects on neuronal structure and behavior. Nat Neurosci 19, 905–914. https://doi.org/10.1038/nn.4315
140. Henkel, N.D., Wu, X., O, S.M., Devine, E.A., Jiron, J.M., Rowland, L.M., Sarnyai, Z., Ramsey, A.J., Wen, Z., Hahn, M.K., McCullumsmith, R.E., 2022. Schizophrenia: a disorder of broken brain bioenergetics. Mol Psychiatry 27, 2393–2404. https://doi.org/10.1038/s41380-022-01494-x
141. Martins-De-Souza, D., Harris, L.W., Guest, P.C., Bahn, S., 2011. The Role of Energy Metabolism Dysfunction and Oxidative Stress in Schizophrenia Revealed by Proteomics. Antioxid Redox Signal 15, 2067–2079. https://doi.org/10.1089/ars.2010.3459
177. Ageta-Ishihara, N., Kinoshita, M., 2021. Developmental and postdevelopmental roles of septins in the brain. Neurosci Res. https://doi.org/10.1016/j.neures.2020.08.006

178. Benoit, B., Poüs, C., Baillet, A., 2023. Septins as membrane influencers: direct play or in association with other cytoskeleton partners. Front Cell Dev Biol 11, 1112319. https://doi.org/10.3389/fcell.2023.1112319
179. Chen, T., Zhou, Li, Yuan, Yue, Fang, Yin, Guo, Yue, Huang, Huizhe, Zhou, Qin, Lv, Xiaoyan, Hammerschmidt, M., Zhou, Q, Fang, Y, Lv, X, Zhou, L, Yuan, Y, Guo, Y, Zhou, : Q, Huang, H, 2014. Characterization of Bbx, a member of a novel subfamily of the HMG-box superfamily together with Cic. Dev Genes Evol 224, 261–268. https://doi.org/10.1007/s00427-014-0476-x
180. Dixon, C., Harvey, T.J., Smith, A.G., Gronostajski, R.M., Bailey, T.L., Piper, M., 2013. Nuclear Factor One X Regulates Bobby Sox During Development of the Mouse Forebrain. Cell Mol Neurobiol 33, 867–73. https://doi.org/10.1007/s10571-013-9961-4
181. Guest, P.C., Iwata, K., Kato, T.A., Steiner, J., Schmitt, A., Turck, C.W., Martins-De-Souza, D., 2015. MK-801 treatment affects glycolysis in oligodendrocytes more than in astrocytes and neuronal cells: Insights for schizophrenia. Front Cell Neurosci 9. https://doi.org/10.3389/fncel.2015.00180
182. Hagag, N., Halegoua, S., Viola, M., 1986. Inhibition of growth factor-induced differentiation of PC12 cells by microinjection of antibody to ras p21. Nature 319, 680–682. https://doi.org/10.1038/319680a0
183. Martins-de-Souza, D., Gattaz, W.F., Schmitt, A., Maccarrone, G., Hunyadi-Gulyás, E., Eberlin, M.N., Souza, G.H.M.F., Marangoni, S., Novello, J.C., Turck, C.W., Dias-Neto, E., 2009. Proteomic analysis of dorsolateral prefrontal cortex indicates the involvement of cytoskeleton, oligodendrocyte, energy metabolism and new potential markers in schizophrenia. J Psychiatr Res 43, 978–986. https://doi.org/10.1016/j.jpsychires.2008.11.006
184. Middleton, F.A., Mirnics, K., Pierri, J.N., Lewis, D.A., Levitt, P., 2002. Gene Expression Profiling Reveals Alterations of Specific Metabolic Pathways in Schizophrenia.
185. Noda, M., Ko, M., Ogura, A., Liu, D., Amano, T., Takano, T., Ikawa, Y., 1985. Sarcoma viruses carrying ras oncogenes induce differentiation-associated properties in a neuronal cell line. Nature 318, 73–75. https://doi.org/10.1038/318073a0
186. Peterson, E.A., Petty, E.M., 2010. Conquering the complex world of human septins: Implications for health and disease. Clin Genet. https://doi.org/10.1111/j.1399-0004.2010.01392.x
187. Sanchez-Dias Az, A., Blanco, M.A., Jones, N., Moreno, S., 2001. HBP2: a new mammalian protein that complements the ®ssion yeast MBF transcription complex. Curr Genet 40, 110–118. https://doi.org/10.1007/s002940100241
188. Stenmark, H., Olkkonen, V.M., 2001. The Rab GTPase family. Genome Biol 2, REVIEWS3007. https://doi.org/10.1186/gb-2001-2-5-reviews3007
189. Werner, B., Yadav, S., 2022. Phosphoregulation of the septin cytoskeleton in neuronal development and disease. Cytoskeleton (Hoboken). https://doi.org/10.1002/cm.21728
